# CONSIDERATIONS FOR SUSTAINING DEMENTIA CARE INTERVENTIONS IN THE COMMUNITY

**DOI:** 10.1093/geroni/igae098.0256

**Published:** 2024-12-31

**Authors:** Sari Shuman

**Affiliations:** RTI International, Research Triangle Park, North Carolina, United States

## Abstract

As community organizations implement dementia care interventions, they are tasked with the day-to-day intricacies of disseminating their chosen program. However, to ensure dementia services and supports remain in the community, they must assess how they can sustain the intervention into the future. The ability to continue dementia programs hinges on careful planning and consideration of several factors. Community organizations need to consider and assess the immediate and longer-term demand for the program in the geographic region being served, determine if they have or need to build organizational support, assess staffing needs and training abilities, and decide how they will continue to fund the dementia services and supports offered through the program if it is successful. In terms of funding, estimating the present and future costs of the dementia care intervention can help community organizations determine if the full program or only portions can be sustained using current or future funding mechanisms. Community organizations also need to secure the commitment from organizational decision makers to continue dementia care programs that have proven effective. Collecting and analyzing program data to demonstrate the program’s impact on participants can assist in garnering support from key organizational stakeholders. In this session, these considerations and others for sustaining dementia care interventions will be presented and discussed.

